# Stromal BMP signaling regulates mucin production in the large intestine via interleukin-1/17

**DOI:** 10.1126/sciadv.adi1827

**Published:** 2023-10-27

**Authors:** Yalong Wang, Ruoyu Lou, Zhe Zhang, Chuyu Xiao, Shicheng Yu, Siting Wei, Yuan Liu, Wei Fu, Baojie Li, Ye-Guang Chen

**Affiliations:** ^1^ The State Key Laboratory of Membrane Biology, Tsinghua-Peking Center for Life Sciences, School of Life Sciences, Tsinghua University, Beijing 100084, China.; ^2^ Guangzhou Institutes of Biomedicine and Health, Chinese Academy of Sciences, Guangzhou 510530, China.; ^3^ Guangzhou National Laboratory, Guangzhou 510005, China.; ^4^ School of Life Sciences, Yunnan University, Kunming 650500, China.; ^5^ Department of General Surgery, Peking University Third Hospital, Beijing 100191, China.; ^6^ Bio-X Institutes, Key Laboratory for the Genetics of Developmental and Neuropsychiatric Disorders, Ministry of Education, Shanghai Jiao Tong University, Shanghai 200240, China.; ^7^ School of Basic Medicine, Jiangxi Medical College, Nanchang University, Nanchang 330031, China.

## Abstract

Bone morphogenic protein (BMP) signaling is critical for intestinal development, homeostasis, and function performance. Although the function of BMP signaling in the intestinal epithelium is well appreciated, the direct effect of BMP on intestinal stromal cells is poorly understood. Here, we show that disruption of BMP signaling by genetic ablation of *Alk3* or *Smad4* expands the stromal cell pool, the mucosa tumefaction, and colonic polyposis in the large intestine. Interleukin (IL) secretion by stromal cells is notably increased, including IL-1, IL-11, and IL-17. Specifically, IL-1 and IL-17a hyperactivate the mucin production by goblet cells through nuclear factor κB signaling, and abnormal mucin accumulation results in the morphological changes, epithelial barrier destruction, and polyposis development. Together, our results provide an insight into the role of BMP signaling in intestinal stromal cells to regulate epithelium function. This study further highlights the role of mucin-producing goblet cells in intestinal homeostasis and colitis development.

## Teaser

Stromal BMP signaling regulates intestinal mucin production via IL-1/17 for intestinal homeostasis and colitis development.

## INTRODUCTION

The intestine is an important organ for food digestion, nutrient absorption, hormone secretion, immune regulation, and host defense (*1*). Its multiple functions are based on the cooperation between intestinal epithelium and mesenchyme. In addition to the immune cells, the intestinal mesenchyme also contains stromal cells, such as the telocytes for Wnt activation and bone morphogenic protein (BMP) inhibition (*2*, *3*), Gli1^+^ cells for Wnt signals (*4*), and Pdgfra^low^/Cd34^+^/Cd81^+^ cells for BMP antagonists (*5*). The signaling cross-talk between stromal cells and the epithelium is fundamental to the balance maintenance between intestinal stem cells (ISCs) and mature epithelial cells, the basics of intestinal homeostasis (*1*).

Two types of cell surface receptors mediate BMP signaling: the type II receptor (BMPRII) and the type I receptors (BMPRIA, also called ALK3, and BMPRIB, also called ALK6). BMP binding enhances receptor complex formation and phosphorylation of type I receptors, which, in turn, phosphorylate and activate Smad1, Smad5, and Smad8 and promote a complex formation with Smad4. The Smad complex is accumulated in the nucleus to regulate gene expression. BMP receptors can also signal through non-Smad pathways, such as p38, c-Jun N-terminal kinase, and phosphatidylinositol 3-kinase to regulate multiple cellular functions (*6*). As an essential niche signal, BMP shows a gradually elevating gradient from the crypt bottom toward the villus compartment, which inhibits the proliferation of ISCs and transient-amplifying (TA) cells in the crypt (*5*, *7*), and promotes the terminal differentiation of enteroendocrine cells along the crypt-to-villus as well as hormone switching (*8*). In addition, BMP drives the top villus cell states, including enterocytes and goblet cells, as loss of its type I receptor *Bmpr1a* reduces top villus gene expression (*9*). Impaired maturation of goblet cells, Paneth cells, and enteroendocrine cells are also observed in mice lacking *Bmpr1a* (*10*). These results demonstrate the vital function of BMP signaling in epithelial homeostasis.

Deregulation of BMP signaling contributes to intestine pathogenesis. Frequent mutations in BMP signaling components including BMPRIA/ALK3 and SMAD4 are identified in juvenile polyposis syndrome that is an inherited disease with a high risk of adenocarcinoma (*11*). Conditional deletion of *Bmpr1a* in mouse intestinal epithelium also induces expansion of ISC compartments, ultimately leading to intestinal adenomas (*7*). In line with these, juvenile polyposis-like phenotype and adenomatous polyps become obvious after ectopic expression of the BMP antagonist noggin (*12*). Moreover, the connection between BMP signaling and colorectal cancer development is evidenced on the treatment of this disease by Simvastatin (*13*).

Intestinal immune homeostasis, such as balanced secretion of interleukin (IL) signals, is critical for the functional performance of the intestinal epithelium, and its deregulation leads to pathological conditions (*14*, *15*). In the homeostatic state, the cytokine IL-10 can support ISC renewal (*16*), while IL-13 can increase tuft cell differentiation and expand the goblet cell pool (*17*, *18*). IL-22, a host defense factor (*19*), not only promotes ISC recovery following damage (*20*) but also protects ISC against DNA damage and malignant transformation (*21*). IL-17a also stimulates secretory cell differentiation, including enteroendocrine cells, Paneth cells, and goblet cells (*19*, *22*). ILs are also involved in intestinal diseases, such as intestinal inflammation, colitis, and colorectal cancer. IL-33 shows a high level in the inflamed lesions of patients with inflammatory bowel disease (IBD) (*23*) and acts as either an inflammatory driver or an alarmin (*24*). IL-23 functions as a pathogenic factor in IBD (*25*), and its inhibition becomes a therapeutic strategy for colitis treatment (*26*). Colitis-induced IL-11 can promote colon carcinogenesis (*27*). IL-1 also promotes chemoradiotherapy resistance and disease progression in colorectal cancer through polarizing cancer-associated fibroblasts (*28*).

The intestinal epithelium is regulated by the underneath niche that contains stromal cells secreting various growth factors. For instance, FOXL1^+^ telocytes located near intestinal stem/TA cells provide a source of Wnt activation and BMP inhibition signals for ISC self-renewal (*3*, *29*). CD90^+^ cells are important for colonic stem cell maintenance by secreting Rspo3, Wnt2b, and Grem1 (*30*). Pericyte-like cells, marked by Cspg4 and overlapped with the Foxl1^+^ population in the mouse stomach and small intestine, can produce Wnt signals (*31*). Stromal cells have been divided into three subpopulations: Pdgfra^high^/Cd34^−^, Pdgfra^low^/Cd34^+^/Cd81^−^, and Pdgfra^low^/Cd34^+^/Cd81^+^ (*5*). Pdgfra^high^/Cd34^−^ cells are mainly located at the villus base and serve as a BMP reservoir, while Pdgfra^low^/Cd34^+^/Cd81^+^ cells are abundant just below the crypts and secrete BMP antagonists. Furthermore, Gli1^+^ mesenchymal cells may be critical for colonic homeostasis as blockage of Wnt secretion from these cells by deletion of *Wntless* leads to loss of stem cells in the colon but not in the small intestine (*4*). In addition, Twist^+^ stromal cells have been shown to participate in colitis pathogenesis by secreting prostaglandin E_2_ (*32*).

In this study, to investigate the function of BMP in intestinal stromal cells, we disrupted BMP signaling in Gli1^+^ stromal cells by ablating *Alk3* and *Smad4* and found that the large intestine showed abnormal morphology, accompanied with severe diarrhea and cyst-like polypus. Abundant accumulation of mucins and epithelial barrier destruction were observed in cyst-like polypus. Furthermore, BMP blockade increased the number of stromal cells and enhanced the expression of several ILs, including IL-1, IL-17, and IL-11. We demonstrated that IL-1 and IL-17 could induce goblet cell differentiation and mucin secretion through nuclear factor κB (NF-κB) signaling, which account for the observed cyst-like morphological changes. Together, our results uncover that BMP signaling in stromal cells is important to regulate goblet cell function and maintain epithelium homeostasis.

## RESULTS

### Gli1^+^ stromal cell–specific ablation of Alk3 and Smad4 causes mucosa tumefaction and promotes goblet cell differentiation in the large intestine

**Fig. 1.**
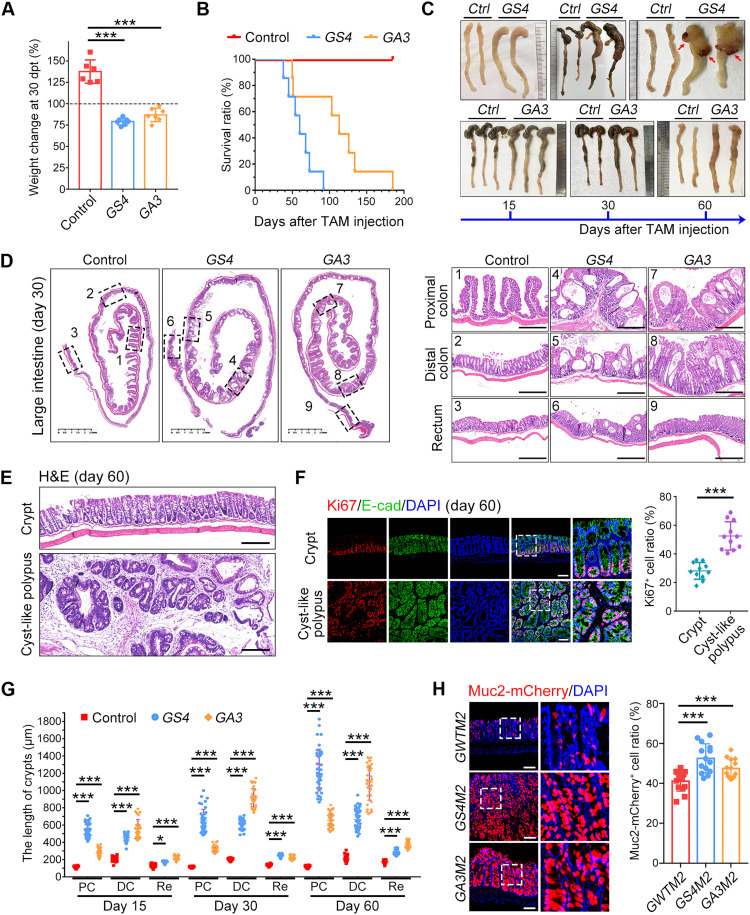
Inactivation of *Smad4* or *Alk3* in Gli1^+^ stromal cells leads to intestinal mucosa tumefaction. (**A**) Body weight changes of indicated mice compared with the starting body weight at day 30 post–tamoxifen administration (dpt). *n* = 6 mice for each group. (**B**) Survival rate of indicated mice after the last tamoxifen (TAM) administration. *n* = 8 mice for each group. (**C**) Representative images of large intestine at different time points after the last tamoxifen administration, and cyst-like polypus appeared at 60 dpt in *GS4* mice (red arrow). Ctrl, control. (**D**) Representative images of hematoxylin and eosin (H&E)–stained large intestine sections and enlarged field of three large intestine segments at 30 dpt. Scale bars, 2.5 mm (left) and 500 μm (right). (**E**) Representative images of H&E-stained cyst-like polypus sections at 60 dpt. Scale bars, 200 μm. (**F**) Ki67 immunofluorescence staining in cyst-like polypus sections from *GS4* mice at 60 dpt. The right panel shows the quantification of Ki67^+^ cells in the cyst-like polypus. Scale bars, 100 μm. DAPI, 4′,6-diamidino-2-phenylindole. (**G**) Crypt length quantification of three large intestine segments along with the time of conditional KO. PC, proximal colon; DC, distal colon; Re, rectum. (**H**) Representative images of Muc2-mCherry in vivo signals in distal colon sections at 30 dpt. The right panel shows the quantification of Muc2-mCherry^+^ cells along the crypt. Scale bars, 100 μm. *P < 0.05 and ***P < 0.001 by one-way analysis of variance (ANOVA) test in (A), (F), (G), and (H).

To explore the function of BMP signaling in stromal cells, we generated stromal-specific heterozygous mice: *Gli1-Cre^ERT2^*;*Smad4^wt/fl^* and *Gli1-Cre^ERT2^*;*Alk3^wt/fl^*. No notable difference was observed on the mouse body weight or the intestinal mucosa morphology in these mice 5 months later after tamoxifen-induced one-allele deletion (fig. S1, A and B). However, compared to control mice (*Gli1-Cre^ERT2^*), homozygous deletion of these genes in *Gli1-Cre^ERT2^;Smad4^fl/fl^* (*GS4*) and *Gli1-Cre^ERT2^*;*Alk3^fl/fl^* (*GA3*) mice resulted in a notably shortened life span, companied with marked body weight loss (Fig. 1, A and B, and fig. S1C). Along with the time, the large intestine was obviously distended, especially in the proximal colon (Fig. 1C). The large intestinal epithelium of BMP deficiency mice showed tumefaction morphology with a cavity structure (Fig. 1D and movies S1 to S3). *GS4* mice also developed cyst-like polypus in 60 days after *Smad4* knockout (KO) (Fig. 1, E and F) (*33*). Moreover, the crypt length of BMP-deleted mice was significantly increased in different segments of the large intestine (Fig. 1G). In addition, both *GS4* and *GA3* mice presented severe diarrhea (fig. S1D). These results suggest that the interruption of BMP signaling in stromal cells evokes morphological and functional disorder in the large intestine.

As Muc2^+^ goblet cells play a critical role in the large intestine by secreting mucus (*34*), we then generated *Gli1-Cre^ERT2^*;*Muc2-mCherry* (*GWTM2*), *Gli1-Cre^ERT2^*;*Smad4^fl/fl^*;*Muc2-mCherry* (*GS4M2*), and *Gli1-Cre^ERT2^*;*Alk3^fl/fl^*;*Muc2-mCherry* (*GA3M2*) mice to examine goblet cell function. As expected, goblet cells in the large intestine of *GS4M2* and *GA3M2* were significantly increased compared with *GWTM2* mice (Fig. 1H). Similar results were obtained in *GA3* and *GS4* mice (fig. S1E), while Ki67^+^ cells and enteroendocrine cells were not changed after *Alk3* or *Smad4* KO (fig. S1, F and G). These results suggest that disruption of BMP signaling in Gli1^+^ stromal cells promotes goblet cell differentiation in the large intestine.

Ablation of *Alk3* and *Smad4* in Gli1^+^ cells had minimal effects on the small intestinal morphology in *GA3* and *GS4* mice (fig. S2A) but increased crypt length in the ileum (fig. S2, B and C). Disruption of BMP signaling in Gli1^+^ cells had no notable effect on Olfm4-marked ISCs (fig. S2D) or goblet cells in the small intestine (fig. S2E). To examine the possible effect on enterocytes, we established *Gli1-Cre ^ERT2^*;*Apoa1-mCherry* (*GWTA1*), *Gli1-Cre ^ERT2^*; *Smad4^fl/fl^*;*Apoa1-mCherry* (*GS4A1*), and *Gli1-Cre ^ERT2^*; *Alk3^fl/fl^*;*Apoa1-mCherry* (*GA3A1*) mice and observed a reduction of enterocytes in the ileum by disruption of BMP signaling in Gli1^+^ cells (fig. S2F). However, Paneth cells showed raised location in ileum in *GA3* mice but not in *GS4* mice (fig. S2G). Abnormal location of Paneth cells in *GA3* mice suggests that Smad4 may mediate other signals to influence Paneth cell maturation.

### Disruption of BMP signaling in Gli1^+^ cells causes mucin accumulation in cavities and the squamous shape of epithelial cells

**Fig. 2.**
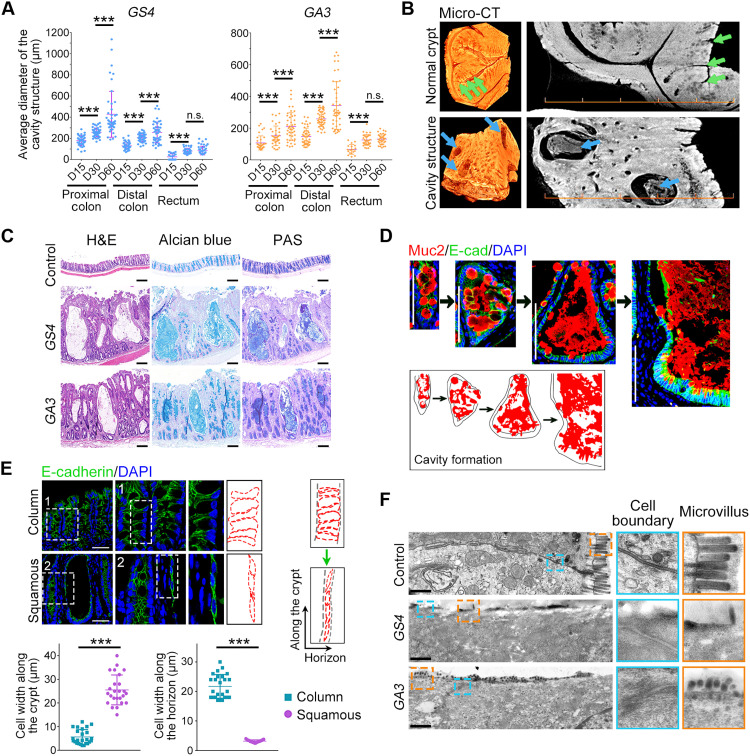
Mucin accumulation results in the cavity formation and the epithelial cell morphology change from column to squamous structure. (**A**) The average diameter of the cavity structure in three large intestine segments of *GS4* and *GA3* mice at different KO times. (**B**) Representative micro-scale computed tomography (micro-CT) images of normal crypts (green arrows) and cavity structures (blue arrows) in the large intestine. Scale bars, 500 μm. (**C**) H&E, Alcian blue, and PAS staining of mucins in the cavities. Scale bars, 200 μm. (**D**) Muc2 and E-cadherin immunofluorescence staining during the cavity development. A sketch depicts an abnormal accumulation of mucins. Scale bars, 100 μm. (**E**) E-cadherin immunofluorescence staining in normal crypts showing the column epithelial cell structure and in cavities showing the squamous epithelial cell structure. The cell width along the crypt and the horizon was quantified (right and bottom). Scale bars, 50 μm. (**F**) Representative transmission electron microscopy images of the large intestinal epithelium and enlarged fields of microvillus and cell boundary. Scale bars, 1 μm. n.s., no significance; ***P < 0.001 by one-way ANOVA test in (A) and (E).

To further characterize the tumefaction phenotype and cavity structures in the large intestine upon BMP blockade in Gli1^+^ stromal cells, we quantified the cavity diameter in three large intestine segments along with the time and observed the cavity enlargement (Fig. 2A). Micro-scale computed tomography revealed that the cavities were filled by protein-like content (Fig. 2B and movies S1 and S2). Alcian blue and periodic acid–Schiff (PAS) staining further confirmed that the cavity was filled with mucins (Fig. 2C), which is consistent with the enrichment of goblet cells upon BMP blockade in Gli1^+^ stromal cells. Along the time, the cavities were filled with abnormal accumulated mucins (Fig. 2D).

We also observed an epithelial morphology change from column to squamous structure accompanied with the cavity expansion based on cell junction staining (Fig. 2E and fig. S3A). In support of this observation, transmission electron microscopy revealed an elongated epithelium and reduced microvillus number along the cavity structure after *Alk3* and *Smad4* depletion (Fig. 2F). Less proliferative cells, goblet cells, and enteroendocrine cells were found in the cavity epithelium (fig. S3B). Together, these results indicate that disruption of BMP signaling in Gli1^+^ stromal cells results in abundant accumulation of mucins in the large intestine to form cavity structures and squamous epithelium with abnormal cell composition.

### Inactivation of BMP signaling in Gli1^+^ cells enhances mucin production in goblet cells

**Fig. 3.**
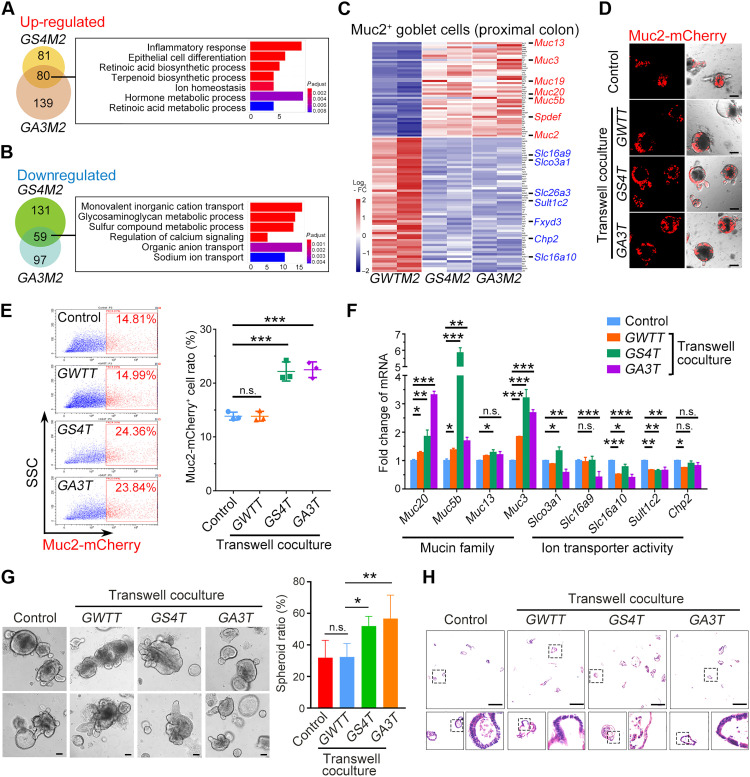
Disruption of BMP signaling in Gli1^+^ stromal cells enhances mucin production and suppresses the ion transport activity in colon goblet cells. (**A**) GO analysis of the up-regulated genes in Muc2-mCherry^+^ cells from *GS4M2* and *GA3M2* mice compared with *GWTM2* mice. (**B**) GO analysis of the down-regulated genes in Muc2-mCherry^+^ cells from *GS4M2* and *GA3M2* mice compared with *GWTM2* mice. (**C**) Heatmap of the up-regulated mucin genes (red) and the down-regulated ion transport genes (blue) in Muc2-mCherry^+^ cells from indicated mice. FC, fold change. (**D**) Representative images of Muc2-mCherry-labeled distal colon organoids cocultured with Gli1-tdTomato^+^ cells from indicated mice for 3 days. Three hundred organoids cocultured with 10,000 Gli1-tdTomato^+^ cells. Scale bars, 100 μm. (**E**) Fluorescence-activated cell sorting (FACS) analysis and quantitation of Muc2-mCherry^+^ cells from distal colon organoids cocultured with Gli1^+^ cells from indicated mice for 3 days. SSC, side scatter. (**F**) Quantitative reverse transcription polymerase chain reaction (qRT-PCR) analysis of mucin and ion transport genes in distal colon organoids cocultured with Gli1^+^ cells from indicated mice for 3 days. (**G**) Representative images of wild-type (WT) distal colon organoids cocultured with Gli1-tdTomato^+^ cells from indicated mice for 3 days and the spheroid ratio quantification. Scale bars, 100 μm. (**H**) Representative images of H&E-stained organoid sections from the coculture system with Gli1-tdTomato^+^ cells from indicated mice for 3 days. Scale bars, 500 μm. *P < 0.05, **P < 0.01, and ***P < 0.001 by one-way ANOVA test in (E) and (G) and two-way ANOVA followed by Tukey’s multiple comparisons test in (F).

To explore the mechanism underlying Muc2^+^ cell enrichment after blocking BMP signaling in Gli1^+^ stromal cells, we examined transcriptome profile in Muc2^+^ cells isolated from *GWTM2*, *GS4M2*, and *GA3M2* mice by RNA sequencing (RNA-seq). Gene ontology (GO) analysis of up-regulated genes in Muc2^+^ cells from *GS4M2* and *GA3M2* mice showed functional associations with inflammatory response, epithelial cell differentiation, and biosynthetic process (Fig. 3A), but down-regulated genes were linked to ion transport and metabolic process (Fig. 3B). Heatmap also showed that mucin-related genes were among the up-regulated genes, while transport activity-related genes were down-regulated (Fig. 3C). These changes mainly took place in the proximal colon (Fig. 3C and fig. S4A), which was in agreement with the more obvious tumefaction phenotype in the proximal colon.

To determine the effect of Gli1^+^ stromal cells on Muc2^+^ goblet cells in vitro, we cocultured primary intestinal Gli1^+^ cells with the organoids derived from distal colon crypts of Muc2-mCherry mice. The *Gli1-Cre^ERT2^*;*Rosa26-tdTomato* (*GWTT*), *Gli1-Cre^ERT2^*;*Smad4^fl/fl^*;*Rosa26-tdTomato* (*GS4T*), and *Gli1-Cre^ERT2^*;*Alk3^fl/fl^*;*Rosa26-tdTomato* (*GA3T*) mice were generated to isolate Gli1^+^ cells. Coculture with Gli1^+^ cells from *GS4T* and *GA3T* mice apparently elevated Muc2-mCherry cells (Fig. 3, D and E). In addition, the expression level of *Alpi*, *Muc2*, and *E-Cad* were increased, but the expression of *Chgb*, *Ki67*, and *Lgr5* was not (fig. S4B), which is consistent with the above observation in the *GS4* or *GA3* intestinal epithelium (Fig. 1H and fig. S1, E to G). Furthermore, the mucin family members were up-regulated, while the ion transport-related genes were down-regulated (Fig. 3F). In addition, the spheroid ratio was increased after coculture with Gli1^+^ cells from *GS4T* and *GA3T* mice (Fig. 3G), the squamous epithelium can also be observed in the cocultured spheroids (Fig. 3H). These results together suggest that BMP signaling blockade in Gli1^+^ stromal cells enhances mucin production and suppresses ion transport in goblet cells, which contributes to the intestinal mucosa tumefaction in the large intestine.

### BMP inhibits cell proliferation and increases IL production of stromal cells

**Fig. 4.**
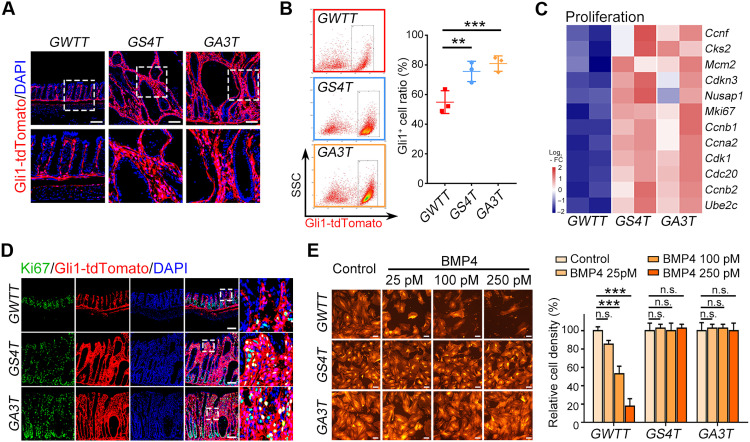
Inactivation of BMP signaling enhances the proliferation of Gli1^+^ stromal cells. (**A**) Representative images of Gli1-tdTomato signals in distal colon sections at 30 dpt. Scale bars, 100 μm. (**B**) FACS analysis and quantitation of Gli1-tdTomato^+^ cells in distal colon from indicated mice at 30 dpt. (**C**) Heatmap of proliferation-related genes in Gli1-tdTomato^+^ cells from the indicated mice at 30 dpt. (**D**) Representative images of Ki67 immunofluorescence staining in distal colon sections of indicated mice at 30 dpt. Scale bars, 100 μm. (**E**) Representative images of WT, Smad4-KO, or Alk3-KO Gli1-tdTomato^+^ cells and the quantification of relative cell density at day 3 after BMP4 treatment. Scale bars, 100 μm. **P < 0.01 and ***P < 0.001 by one-way ANOVA test in (B) and (E).

To better understand the mechanism in which BMP acts on Gli1^+^ stromal cells to influence epithelium homeostasis, we assessed the change of Gli1^+^ stromal cells. Our results showed that the Gli1^+^ stromal cell number was notably increased in the colon after ablation of *Alk3* or *Smad4* (Fig. 4, A and B, and fig. S5A), while no notable changes were observed in the small intestine (fig. S5B). Consistently, Ki67^+^ stromal cells and proliferation genes were increased after *Alk3* or *Smad4* KO (Fig. 4, C and D, and fig. S5C). In addition, BMP treatment inhibited the proliferation of wild-type Gli1^+^ cells but not that of *Alk3* or *Smad4* KO cells (Fig. 4E). These results suggest that BMP inhibits cell proliferation to balance the stromal cell pool, while loss of BMP expands stromal cells and leads to microenvironment turbulence.

**Fig. 5.**
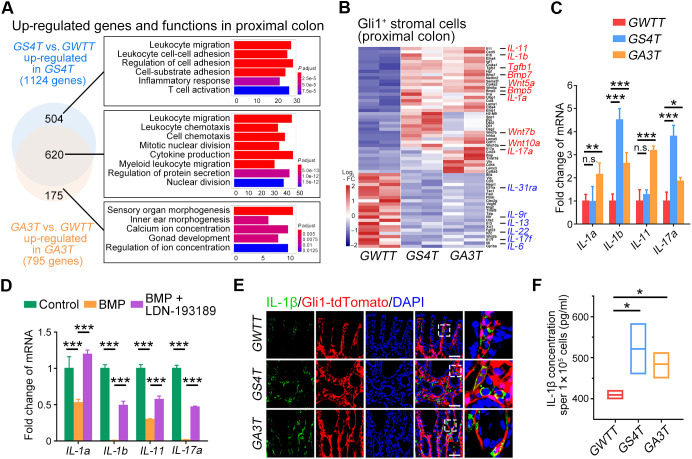
Inactivation of BMP signaling increases IL secretion in Gli1^+^ stromal cells. (**A**) GO analysis of the up-regulated genes in Gli1-tdTomato^+^ cells from *GS4T* and *GA3T* mice compared with *GWTT* mice. (**B**) Heatmap of the up-regulated (red) and down-regulated genes (blue) in Gli1-tdTomato^+^ cells from indicated mice. (**C**) qRT-PCR analysis of IL genes detected in Gli1-tdTomato^+^ cells from indicated mice. (**D**) qRT-PCR analysis of IL genes detected in WT Gli1-tdTomato^+^ cells at day 3 after the treatment with 25 pM BMP4 or 1 μM LDN-193189. (**E**) Representative images of IL-1β immunofluorescence in distal colon sections of indicated mice at 30 dpt. Scale bars, 100 μm. (**F**) Elisa quantitation of IL-1β levels from in vitro cultured Gli1-tdTomato^+^ cells. *P < 0.05, **P < 0.01, and ***P < 0.001 by two-way ANOVA followed by Tukey’s multiple comparisons test in (C) and (D) and one-way ANOVA test in (F).

To assess how Gli1^+^ stromal cells affect the epithelium after BMP signaling disruption, we performed RNA-seq and GO analysis of Gli1-tdTomato^+^ cells. The results showed that commonly up-regulated genes upon *Alk3* or *Smad4* KO were involved in leukocyte migration, cell division, and cytokine production (Fig. 5A and fig. S6A). By analysis of cytokines, we found that IL genes, such as *IL-1*, *IL-17*, and *IL-11*, were up-regulated upon *Alk3* or *Smad4* KO, while *IL-6*, *IL-13*, and *IL-22* were down-regulated (Fig. 5B and fig. S6B). Increased mRNA expression of *IL-1a*, *IL-1b*, *IL-11*, and *IL-17a* was confirmed in cultured *Alk3* or *Smad4* KO Gli1^+^ stromal cells (Fig. 5C), and BMP specifically suppressed their expression (Fig. 5D). Higher protein levels of IL-1α and IL-1β were detected in Gli1^+^ stromal cells of intestinal tissues (Fig. 5E and fig. S6C). Increased skipped exon of *IL-1b* was found in Gli1^+^ stromal cell after *Alk3* or *Smad4* KO (fig. S6D). An increased IL-1β protein level was found in the conditional medium of *Alk3* KO or *Smad4* KO Gli1^+^ cells (Fig. 5F). These results indicate that BMP stimulates expression of *IL-1a*, *IL-1b*, *IL-17a*, and *IL-11* in Gli1^+^ stromal cells.

### IL-1 and IL-17 promote goblet cell differentiation and enhance mucin secretion through NF-κB signaling

**Fig. 6.**
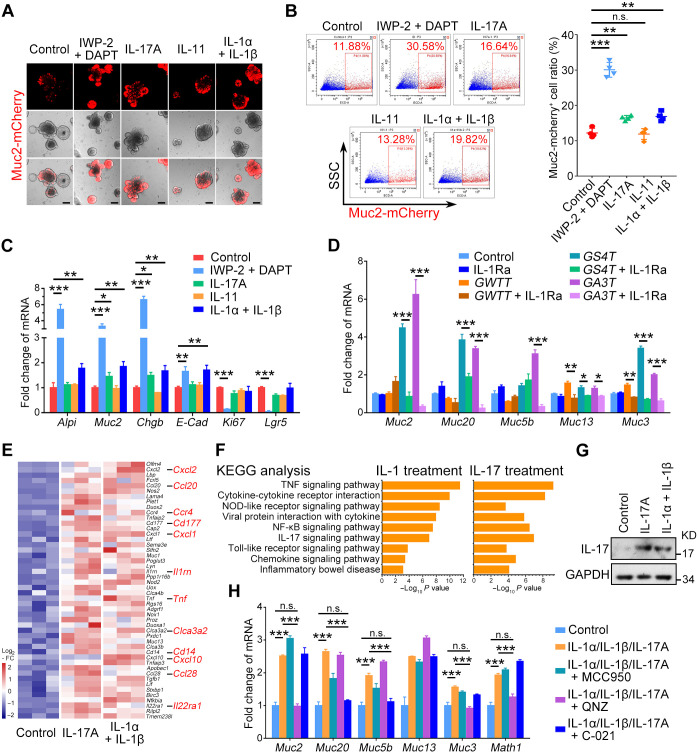
IL-1 and IL-17 promote goblet cell differentiation and enhance mucin production in mouse colonic organoids through NF-κB signaling. (**A**) Representative images of Muc2-mCherry-labeled distal colon organoids at day 3 after the treatments with 2 μM IWP-2, 10 μM DAPT, or 10 nM ILs. Scale bars, 100 μm. (**B**) FACS analysis and quantitation of Muc2-mCherry^+^ cells from distal colon organoids at day 3 after the treatments with 2 μM IWP-2, 10 μM DAPT, or 10 nM ILs. (**C**) qRT-PCR analysis of epithelial marker genes in distal colon organoids at day 3 after the treatments with 2 μM IWP-2, 10 μM DAPT, or 10 nM ILs. (**D**) qRT-PCR analysis of mucin genes in distal colon organoids cocultured with Gli1-tdTomato^+^ cells from indicated mice and treated with 50 nM IL-1Ra for 3 days. (**E**) Heatmap of the up-regulated genes in distal colon organoids after the treatments with 10 nM ILs for 12 hours. (**F**) Kyoto Encyclopedia of Genes and Genomes (KEGG) analysis for the up-regulated signaling pathways after the indicated treatments for 12 hours. (**G**) Immunoblot analysis of the expression level of IL-17 in distal colon organoids treated with 10 nM ILs for 3 days. (**H**) qRT-PCR analysis of mucin genes in distal colon organoids after the indicated treatments for 3 days. *P < 0.05, **P < 0.01, and ***P < 0.001 by one-way ANOVA test in (B) and two-way ANOVA followed by Tukey’s multiple comparisons test in (C), (D), and (H).

To investigate the effect of ILs on goblet cells, we treated colonic organoids derived from the crypts of Muc2-mCherry mice with IL-1, IL-11, and IL-17. Inhibition of Wnt and Notch signaling with IWP-2/DAPT has been shown to promote goblet cell differentiation (*35*). IWP-2/DAPT greatly enhanced Muc2-mCherry^+^ cells (Fig. 6, A and B). Notably, IL-17 or IL-1, but not IL-11, also increased Muc2-mCherry^+^ cells (Fig. 6, A and B). Consistently, the goblet cell marker genes were increased upon IL-1 or IL-17 treatment (Fig. 6C and fig. S7A). Conversely, IL-1 receptor antagonist (IL-1Ra) suppressed mucin mRNA levels in colonic organoids cocultured with *Alk3* or *Smad4* KO Gli1-tdTomato^+^ cells (Fig. 6D). IL-1 and IL-17 also enhanced mucin expression in small intestinal organoids (fig. S7, B to D). Mucin accumulation or cavity structures were only found in the large intestine but not in the small intestine of *Alk3* or *Smad4* KO mice, perhaps due to more Gli1^+^ cells in the colon (*4*).

Gli1^+^ stromal cells also secrete BMP ligands, such as BMP5 and BMP7 (Fig. 5B and fig. S6B), which may contribute to goblet differentiation (*9*, *10*). To investigate the influence of BMP on goblet cells, we added the BMP receptor inhibitor LDN-193189 in the colonic organoids and Gli1^+^ stromal cell coculture system. As shown in fig. S7E, LDN-193189 increased *Lgr5* and *Alpi* expression but did not suppress the increased expression of *Muc2*. These results together suggest that BMP suppresses IL expression in Gli1^+^ stromal cells to control goblet cell differentiation.

To explore how IL-1/17 affect the intestinal epithelium, RNA-seq was performed after the organoids were treated for 12 hours. Chemokines and cytokines were up-regulated by IL-1 or IL-17 (Fig. 6E and fig. S7F). These increased genes involved in tumor necrosis factor (TNF) signaling, nonobese diabetic (NOD)–like signaling, NF-κB signaling, and cytokine-cytokine receptor interaction, as revealed by Kyoto Encyclopedia of Genes and Genomes (KEGG) analysis (Fig. 6F). IL-1/17 treatment could increase IL-17 expression in colon organoids (Fig. 6G and fig. S7G). To define the pathways involved in IL-1/17 signaling, three inhibitors were used: QNZ (EVP4593) for NF-κB signaling and TNF-α production, MCC950 for NOD-like signaling, and C-021 for CCR4 signaling. We found that QNZ inhibited the increased expression of *Muc2* and *Math1* (a transcript factor for goblet differentiation), while C-021 partly impaired the increased expression of *Muc20* and *Muc5b* induced by IL-1 or IL-17 (Fig. 6H). These results suggest that IL-1 and IL-17 promote goblet cell differentiation and enhance mucin secretion through NF-κB signaling and CCR4 signaling.

To further explore the role of IL-1/17, RNA-seq and gene expression analysis were performed after the organoids were treated with IL-1 or IL-17 for 3 days. Mucins and solute carrier (SLC) family members were regulated by IL-1 and IL-17, including the increased expression of *Muc1* and *Muc13* and the decreased expression of *Slc16a10* and *Chp2* (fig. S7H). The decreased expression of ion transport-related genes *Slc16a10* (MOT10) and *Chp2* (CHP2) were further confirmed at mRNA and protein levels (fig. S7, I and J), which was consistent with the above results (Fig. 3F). GO analysis also revealed that IL-1 and IL-17 up-regulated the genes related to regulation of cell morphogenesis, cell shape, and mucus secretion while decreasing the ones related to regulation of mitotic cell cycle, cellular amino acid metabolic process, and cilium organization (fig. S7K).

### IL-1 signals are increased in human colitis and enhance mucin secretion in human colonic organoids

**Fig. 7.**
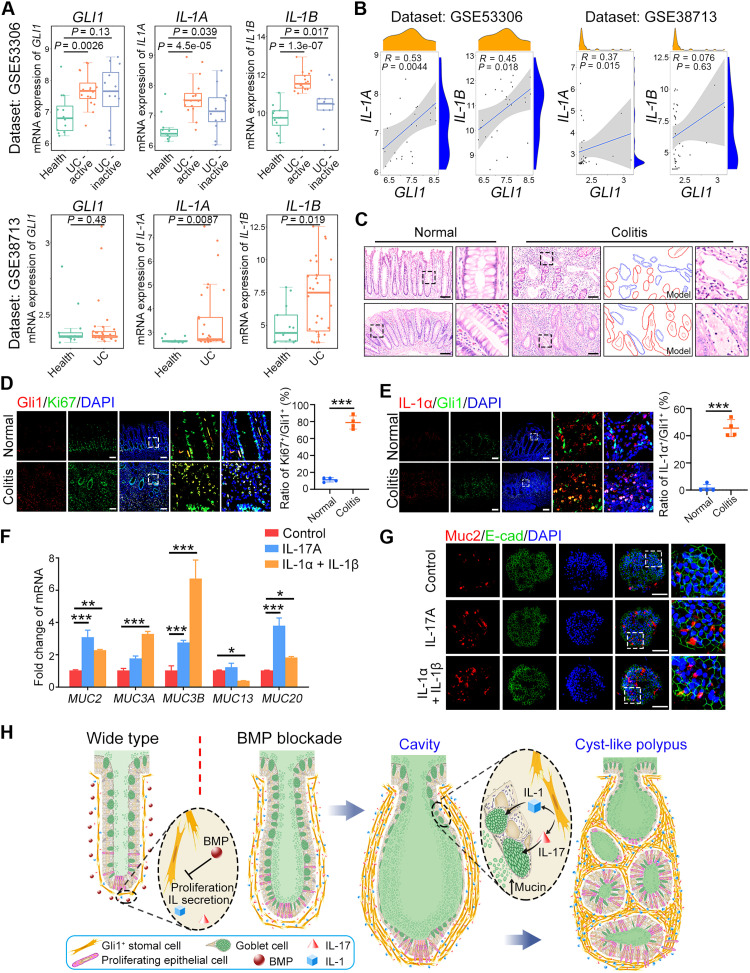
Increased IL-1 signals enhance the mucin secretion in human colitis samples. (**A**) Box plots of mRNA levels of GLI1 and ILs in healthy and colitis specimens (using datasets GSE53306 and GSE38713). In the box plots, the middle line depicts the median and the whiskers in the min-to-max range. UC, ulcerative colitis. (**B**) The positive correlation between *GLI1* and *IL-1A* or *IL-1B* in human datasets GSE53306 and GSE38713. (**C**) Representative images of H&E staining in human large intestine and colitis sections. The red lines show the column epithelial cell structure and the blue lines show the squamous epithelial cell structure in colitis model. Scale bars, 100 μm. (**D**) Gli1 and Ki67 immunostaining and quantification of human colon derived from healthy and ulcerative colitis specimens. Scale bars, 100 μm. (**E**) IL-1α and Gli1 immunostaining and quantification of human colon derived from healthy and ulcerative colitis specimens. Scale bars, 100 μm. (**F**) qRT-PCR analysis of mucin genes in human large intestinal organoids after treated with 10 nM IL-1 or IL-17 for 3 days. (**G**) Representative images of Muc2 and E-cadherin immunofluorescence costaining of human colon organoids after treated with IL-1 or IL-17 for 3 days. Scale bars, 100 μm. (**H**) A working model shows that disruption of BMP signaling in Gli1^+^ stromal cells increases goblet cell number and promotes mucin production through IL-1 and IL-17. *P < 0.05, **P < 0.01, and ***P < 0.001 by one-way ANOVA test in (D) and (E) and two-way ANOVA followed by Tukey’s multiple comparisons test in (F).

It has been reported that GLI1 is associated with colitis and promotes colitis-associated tumorigenesis (*36*). Our KEGG analysis also indicated that IL-1 and IL-17 might function in IBD pathogenesis (Fig. 6F). We then asked whether the regulation of goblet cells by IL from stromal cells contributes to human colitis development. We first analyzed the expression levels of *GLI1*, *IL-1A*, and *IL-1B* with the published dataset (*37*, *38*) and found that they were increased in ulcerative colitis compared to health tissues (Fig. 7A). *GLI1* expression was positively correlated with *IL-1A* and *IL-1B* in these datasets (Fig. 7B). Similar to the *Alk3* KO and *Smad4* KO mice (Fig. 2C), cavity structures filled with mucins were also observed in human colitis samples (Fig. 7C and fig. S8A). Ki67 signals in GLI1^+^ cells was increased in human colitis (Fig. 7D), so as the secretion of IL-1α and IL-1β (Fig. 7E and fig. S8B). IL-1β expression was also increased in GLI1^+^ cells in human colorectal cancer (fig. S8C). In addition, cytokines and inflammatory response were also up-regulated in the dextran sodium sulfate (DSS)–induced mouse colitis model, such as the CXC subfamily, CC subfamily and IL-1 signaling (fig. S8D). The increased protein level and positive cell ratio of IL-1α or IL-1β were observed in DSS-induced colitis (fig. S8, E and F). The increased colocalizations of GLI1 with IL-1β or Ki67 were also detected (fig. S8, G and H). Decreased p-Smad1/5 level was observed in human colitis and DSS-induced colitis (fig. S8, I and J) (*39*), indicating that the inactivation of BMP signaling may account for high proliferation capacity of GLI1^+^ cells shown by up-regulated Ki67 signals. Furthermore, IL-17 and IL-1 also up-regulated mucin gene expression and Muc2 protein level in human colonic organoids (Fig. 7, F and G). These data indicate that high IL levels may contribute to pathogenesis of human colitis.

## DISCUSSION

The interplay between intestinal epithelium and stromal cells is critical for intestinal functionality and homeostasis (*40*), and dysfunction of any of them can drive pathological development (*40*, *41*). However, most studies in the past focus on polyposis initiation and tumorigenesis due to dysfunction of BMP signaling in the intestinal epithelium (*5*, *42*). It is unclear whether BMP regulates stromal cell activity and whether stromal BMP signaling plays a role in intestinal epithelium homeostasis. Here, we demonstrate that *Alk3* or *Smad4* KO in Gli1^+^ cells results in tumefaction, cavity formation, and polyposis development in the large intestine. We further show that interruption of BMP signaling provokes a high production of IL-1 and IL-17, which stimulate abnormal mucin secretion in goblet cells (Fig. 7H).

Goblet cells secrete mucins to form mucous layers, which stabilize the bacterial biofilm and prevent the epithelium from pathogen invasion (*34*). The destruction of mucosal barrier and imbalance of mucin dynamics may lead to enteritis development (*43*). For example, reduction in goblet cell number, impairment of mucin secretion and shrinkage of mucus layer are associated with IBD in human and colitis in mouse model (*44*, *45*). However, consequences initiated by the abnormal accumulation of mucins are obscure. Our results show that excessive mucin production accounts for the formation of cavity structure, which further results in epithelium tumefaction and changes the epithelial morphology from column to squamous structure. Goblet cell emptying can be found in normal mucus secretion, and the column structure can be recovered from membrane recombination and replenishment of mucin granules (*46*, *47*). We hypothesize that persistent mechanical force produced by the cavity structure prevents the column-structure recovery and maintains the squamous structure. That abnormal epithelial morphology and barrier are associated with human ulcerative colitis and DSS-induced mouse colitis may induce the regenerative and proliferative progress of the intestinal epithelium and result in the colonic polyposis development (*48*–*51*).

Gli1, a transcription factor acting downstream of Hedgehog signaling (*52*), can label a subgroup of mesenchymal cells that produce Wnt ligands and contribute to the maintenance of colon stem cells (*4*). In addition to growth factors, cytokines and chemokines produced from stromal cells are also pivotal intestinal niche factors to regulate epithelium activity (*53*). However, regulation on these cytokine or chemokine-secreted stromal cells is not fully understood. Our results revealed that BMP signaling suppresses the expansion of Gli1^+^ stromal cells and IL production, which is critical for appropriate homeostatic control of goblet cells in their amount and their mucin-secreting activity. However, how BMP signaling regulates Gli1^+^ stromal cells requires further exploration.

The mucosa tumefaction, abnormal cavity structures, and mesenchymal cell overexpansion have been reported in the large intestine after the KO of *Alk3* in Foxl1- or Col1a2-positive cells, which may be due to intestinal immune and extracellular microenvironment and CXCL12 signaling (*54*, *55*). In addition to these phenotypes, we also observed that the abnormal mucin accumulation was associated with cavity formation and the epithelial squamous shape transformation in the mice with *Alk3* or *Smad4* KO in Gli1^+^ cells. We further found that IL-1/17 from Gli1^+^ cells may account for enhanced goblet differentiation and mucin secretion. These results suggest that disruption of BMP signaling in intestinal stromal cells can remodel the intestinal microenvironment and regulate the epithelial cell fate by regulating cytokine production.

IL-1 can induce gastrointestinal inflammation (*56*), promote colitis and tumor development (*57*), and associate with therapy nonresponse and unfavorable tumor prognosis (*57*, *58*). Most of these functions of IL-1 are associated with immune cells. It is not known whether IL-1 can directly regulate epithelial cells. We found that IL-1 promotes mucin secretion in goblet cells and leads to the excessive mucin accumulation. NF-κB signaling and CCR4 signaling may contribute to these processes. Nonetheless, our results uncover a previously unknown link of subepithelial stromal cells to control goblet cell function in the intestinal epithelium and provide insights into development of therapeutic treatment for intestinal colitis.

## MATERIALS AND METHODS

### Mice and ethics statement

*Gli1-Cre^ERT2^* mice were from L. Baojie (*59*); *Alk3^fl/fl^* mice were provided by Y. Mishina (*60*); *Smad4^fl/fl^* mice were provided by Y. Xiao (*61*); *Rosa26-Loxp-STOP-Loxp-tdTomato* mice were obtained from the Jackson Laboratory; *Muc2-mCherry* mice and *Apoa1-mCherry* mice were commercially generated by Gem Pharmatech (Nanjing). Both male and female mice ranging from 2 to 4 months old in age were used. No statistical method was used to predetermine sample size. In general, we used at least three mice per genotype in each experiment. For Cre induction, mice were intraperitoneally injected with 100 μl of tamoxifen dissolved in oil at 20 mg/ml for five consecutive days. All animal studies were performed in accordance with the guidelines and under the approval of the Institutional Animal Care and Use Committee of Tsinghua University (YGC-19).

### Isolation of mouse intestinal crypts and organoid culture

Mouse intestine was split longitudinally and washed three times with cold phosphate-buffered saline (PBS). Villi were carefully scraped away, and small pieces (5 mm) of intestine were incubated in 0.5 mM EDTA in PBS for 30 min at 4°C. These pieces were then vigorously suspended in cold PBS, and the mixture was passed through 70-μm cell strainer (BD Biosciences). Large intestine was directly scraped after incubated in 0.5 mM EDTA in PBS for 30 min at 4°C. The crypts were enriched through centrifugation (3 min at 1000 rpm). Then, the crypts were embedded in Matrigel (BD Biosciences) and seeded on 24-well plate. Small intestinal organoids were cultured as previously described (*7*), and additional CHIR-99021 (2.5 μM; Selleck) was added into large intestinal organoids. For chemical treatments, IWP-2 (2 μM; Selleck), DAPT (10 μM; Selleck), or indicated ILs (10 nM; Novoprotein) were added to the cultured medium. After 3 or 6 days, RNA or single-cell suspension was selected for quantitative reverse transcription polymerase chain reaction (qRT-PCR) or fluorescence-activated cell sorting (FACS) analysis, respectively.

### Patient-derived colon tissues and organoid culture

The human materials used in this study have been previously described (*62*). Briefly, intestine tissues were surgically resected and sampled from patients who had been diagnosed with intestine tumors at the Peking University Third Hospital, Beijing, China. All tissue samples were obtained with informed consent, and the study was approved by the Peking University Third Hospital Medical Science Research Ethics Committee (M2018083). The human colon organoids were cultured as previously described (*62*).

### Isolation and culture of mesenchymal cells

Mouse large intestine was split longitudinally and trimmed into small pieces. After shocked in cold PBS vigorously, tissues were transferred into cold PBS with 1 mM DTT and incubated in 37°C shaker (10 min at 250 rpm). Then, tissues were transferred into cold PBS with 0.5 M EDTA and 1 M Hepes and incubated in 37°C shaker for two times (10 min at 250 rpm), which was followed by digestion with collagenase IV (10,000 U/ml; Gibco) in RPMI 1640 medium (Gibco) and incubated in 37°C incubator for 45 min. Last, tissue pieces were then vigorously suspended in culture medium containing 10% fetal bovine serum (ExCell) and 1% penicillin-streptomycin (HyClone) in MEM alpha basic medium (Gibco), and the mixture was passed through 70-μm cell strainer (BD Biosciences). The cell suspension was added into culture medium and cultured in 10-cm dishes.

### Immunohistochemistry and immunofluorescence staining

Intestines isolated from the indicated mice were fixed with 4% formaldehyde solution for overnight and dehydrated with gradient concentration ethanol. Then, tissues were embedded in paraffin with paraffin embedding machine (Arcadia H+C; Leica), and sections were prepared with paraffin slicing machine (RM2255; Leica). For frozen sections, intestine tissues were fixed with 4% formaldehyde solution for 2 hours at 4°C, followed by dehydrating in 30% sucrose solution at 4°C overnight. Next, the tissues were embedded in optimal cutting temperature compound (Sakura) and stored at −80°C. Sections were prepared with freezing slicing machine (CM1950; Leica). For staining, the sections were permeabilized with PBST solution (3% bovine serum albumin and 0.1% Triton X-100 in PBS) and then incubated overnight with the primary antibody at 4°C. The fluorescein-labeled secondary antibodies (1:800; the Jackson Laboratory) for immunofluorescence or secondary horseradish peroxidase–conjugated goat anti-rabbit antibody (ZSGB-BIO) for immunohistochemistry were added for 2 hours at room temperature. Confocal laser scanning (FV3000; Olympus) or 3,3′-diaminobenzidine chromogenic fluid (ZSGB-BIO) was used to detect the staining signals.

### Chemical staining

Intestine tissue sections were rehydrated in gradient concentration ethanol and PBS. For hematoxylin and eosin staining, hematoxylin (ZSGB-BIO) was added onto slides and covered tissue region for 2 min, followed by washing in a large amount of water. Then, section was covered by eosin (Beyotime) for 2 min and washed by water flow. For mucin staining, Alcian Blue & Nuclear Fast Red Staining Kit (BASO) was carried out according to the manufacturer’s instructions. For PAS staining, the AB-PAS Staining Kit (Solarbio) was used. In addition, the BCIP/NBT Alkaline Phosphatase Color Development Kit (Beyotime) was performed for alkaline phosphatase staining. The sections were dehydrated and sealed by neutral gum (ZSGB-BIO) for 48 hours, and sections were scanned by a pathological section scanner (KF-PRO-120; KFBIO).

### Antibodies

Rabbit anti-Ki67 (1:300; ab15580, Abcam), rabbit anti-Mucin 2 (1:300; ab272692, Abcam), rabbit anti-lysozyme (1:300; ab108508, Abcam), rabbit anti-chromogranin A (1:300; ab15160, Abcam), mouse anti–E-cadherin (1:1000; 610182, BD Biosciences), mouse anti–α-catenin (1:500; 610194, Biosciences), mouse anti–β-catenin (1:500; 610154, Biosciences), rabbit anti-BMPR1A (1:1000; sc-20736, Santa Cruz Biotechnology), rabbit anti-Smad4 (1:1000; sc7154, Santa Cruz Biotechnology), rabbit anti-Olfm4 [1:300; 39141, Cell Signaling Technology (CST)], mouse anti-vimentin (1:500; 550513, BD Biosciences), rabbit anti–IL-1α (1:50; ab300499, Abcam), rabbit anti–IL-1α (1:50; 16765-1-AP, Proteintech), mouse anti–IL-1β (1:200; sc-52012, Santa Cruz Biotechnology), rabbit anti-MOT10 (1:1000; YT7177, ImmunoWay), rabbit anti-CHP2 (1:1000; YT0915, ImmunoWay), rabbit anti–p-Smad1/5 (1:100; 9516, CST), mouse anti-Ki67 (1:200; 9449, CST), and rabbit anti-Gli1 (1:80; 3538, CST).

### FACS analysis

For stromal cells isolated from mice, dissociated cells were passed through 40-μm cell strainer (BD Biosciences), and single tdTomato positive cells were sorted by flow cytometry (MoFlo Astrios EQ; Beckman). For cultured organoids derived from *Muc2-mCherry* mice, organoids embedded in Matrigel were suspended in cold PBS after medium removal and pelleted by centrifugation (3 min at 1000 rpm). The organoids were dissociated in TrypLE (Invitrogen) for 15 min at 37°C and passed through 40-μm cell strainer, and single Muc2-mCherry positive cells were sorted by flow cytometry (CytoFLEX S; Beckman).

### qRT-PCR analysis

The cultured organoids were precipitated, and RNA was purified using the RNeasy Kit (mf167-01, Mei5 Biotech) and converted into cDNA using the NovoScript One-Step RT-PCR kit (Novoprotein). Real-time PCR reactions were performed in triplicates on a LightCycler 480 (Roche). Primers used were listed in table S1.

### Bulk RNA-seq and GO and KEGG analysis

RNA-seq was performed with the Illumina sequencing platform, and the quality assessment was done by FastQC (version 0.11.9). Then, the data were uniquely mapped to the mm10 genome by STAR (version 2.5.3), and the normalized gene counts were performed by DESeq2 (version 1.28.1) (*63*, *64*). The analysis of differentially expressed genes (DEGs) and expression values was performed by DESeq2 (version 1.28.1) software (*64*). Genes with absolute log_2-_transformed fold changes ≥ 1 were regarded as DEGs, and a threshold of *P* value < 0.05 was used. The primary analysis data of Muc2-mCherry^+^ cells and Gli1-tdTomato^+^ cells are presented in tables S2 and S3. The normalized gene expression data of distal colon organoids treated with IL-1 or IL-17 for 12 or 72 hours are presented in table S4. The heatmap of gene fold changes was generated by Pheatmap (version 1.0.12). The intersection analysis of differential genes of each component was displayed in the VennDiagram (version 1.7.3). DEGs’ GO enrichment and KEGG analysis were carried out by ClusterProfiler (version 3.16.1) (*65*).

### ELISA analysis

For in vitro cultured Gli1^+^ cells, 1 × 10^5^ cells were cultured for 24 hours. After the medium was discarded, the cells were washed twice with preheated PBS, and MEM-α medium without serum or phenol red (Gibco) was used for further culture. One day later, the culture medium was collected for IL-1β enzyme-linked immunosorbent assay (ELISA) detection according to the manufacturer’s instructions (RK00006, ABclonal).

### Gene expression analysis from colitis datasets

Microarray data were derived from Gene Expression Omnibus (GEO) dataset [GSE38713 (*37*) and GSE53306 (*38*)]. The GEO datasets were directly accessed through the R (v4.2.1) package GEOquery to retrieve normalized expression data (*66*). Then, the R (v4.2.1) package ggpubr (https://cran.r-project.org/web/packages/ggpubr/index.html) was used to generate box plots and calculate respective *P* value, and Wilcoxon rank sum tests were used to compare two conditions.

### Statistics

All experiments were performed independently at least three times with three replicates. Data shown in column and dot graphs represent means ± SD. Statistical differences were analyzed using one-way analysis of variance (ANOVA) test or two-way ANOVA followed by Tukey’s multiple comparisons test. *P < 0.05, **P < 0.01, and ***P < 0.001. All statistical analysis was performed with GraphPad Prism 8 (Windows 10).
