# Supplementary material for: Stromal BMP signaling regulates mucin production in the large intestine via interleukin-1/17
Source: Sci Adv. 2023 Oct 27;9(43):eadi1827. doi: 10.1126/sciadv.adi1827 (PMC10610902; doi:10.1126/sciadv.adi1827)
Supplement: Supplementary file 1 — Figs. S1 to S8 Legends for tables S1 to S4 Legends for movies S1 to S3 [file sciadv.adi1827_sm.pdf]

Science Advances
AAAS

Supplementary Materials for

Stromal BMP signaling regulates mucin production in the large intestine via interleukin-1/17

Yalong Wang *et al.*

Corresponding author: Ye-Guang Chen, ygchen@tsinghua.edu.cn

Sci. Adv. **9**, eadi1827 (2023)
DOI: 10.1126/sciadv.adi1827

The PDF file includes:

Figs. S1 to S8
Legends for tables S1 to S4
Legends for movies S1 to S3

Other Supplementary Material for this manuscript includes the following:

Tables S1 to S4
Movies S1 to S3

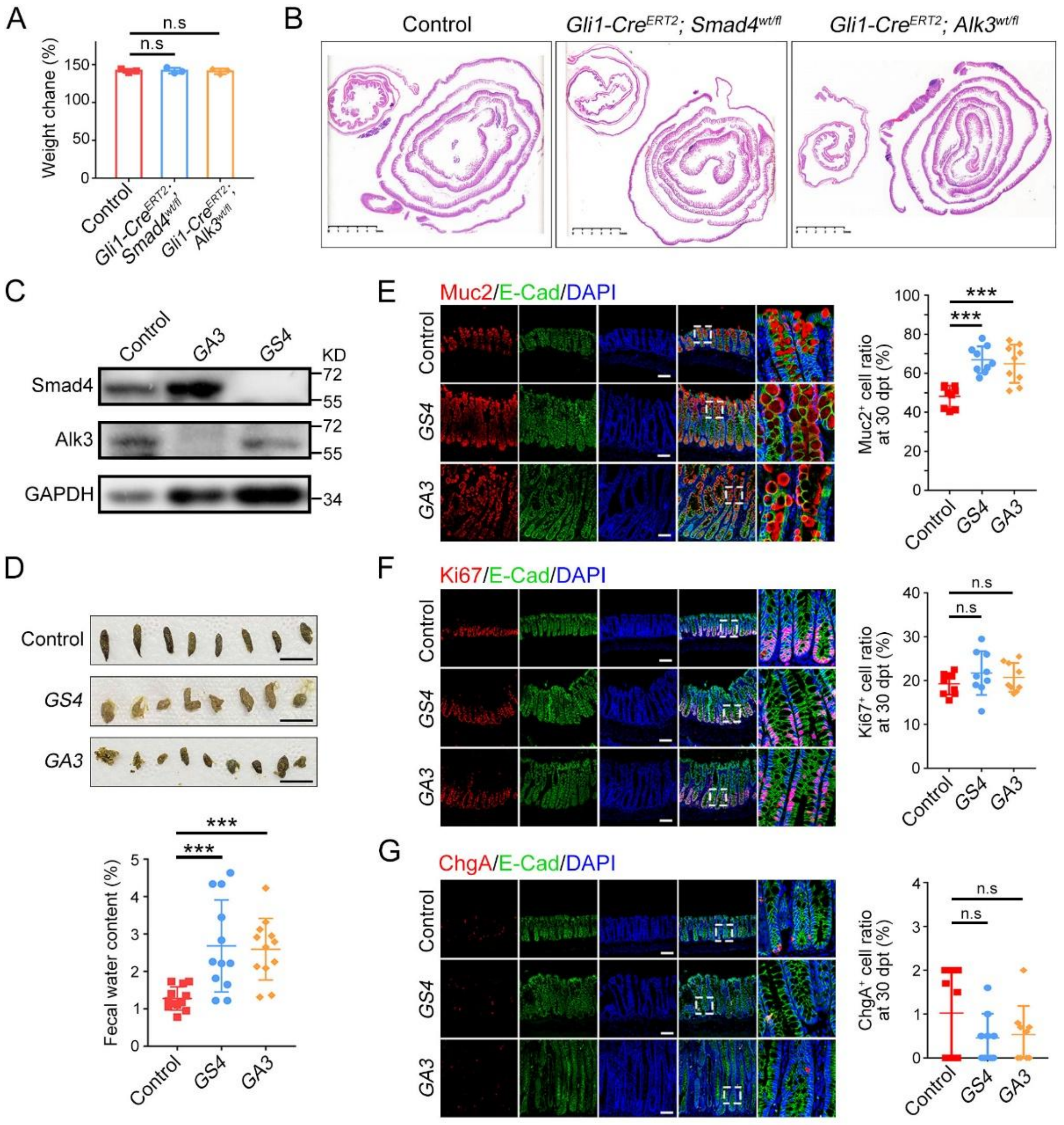

Fig. S1. Stromal-specific *Smad4*- or *Alk3*-knockout mice exhibit severe diarrhea.

(A) Quantification of body weight change in control and stromal-specific heterozygous *Smad4*- or *Alk3*-knockout mice compared with the starting body weight at day 150 post tamoxifen administration (dpt). N = 3 mice for each group.

(B) Representative images of H&E-stained intestine sections from control and heterozygous *Smad4*- or *Alk3*-knockout mice at 150 dpt. Scale bars: 5 mm.

(C) Immunoblot of *Smad4* and *Alk3* proteins in Gli1^{+} stromal cells from the indicated mice at 30 dpt.

(D) The feces from the indicated mice at 30 dpt, and the quantification of water content

(bottom). Scale bars: 1 cm.

(E to **G)** Muc2 (E), Ki67 (F) and ChgA (G) immunofluorescence staining of distal colon sections from the indicated mice at 30 dpt, and the positive cell ratio along the crypt was calculated against the total cell number. Scale bars: 100 μm.

n.s: no significance, ***P<0.001 by one-way ANOVA test in (A), (D), (E), (F) and (G).

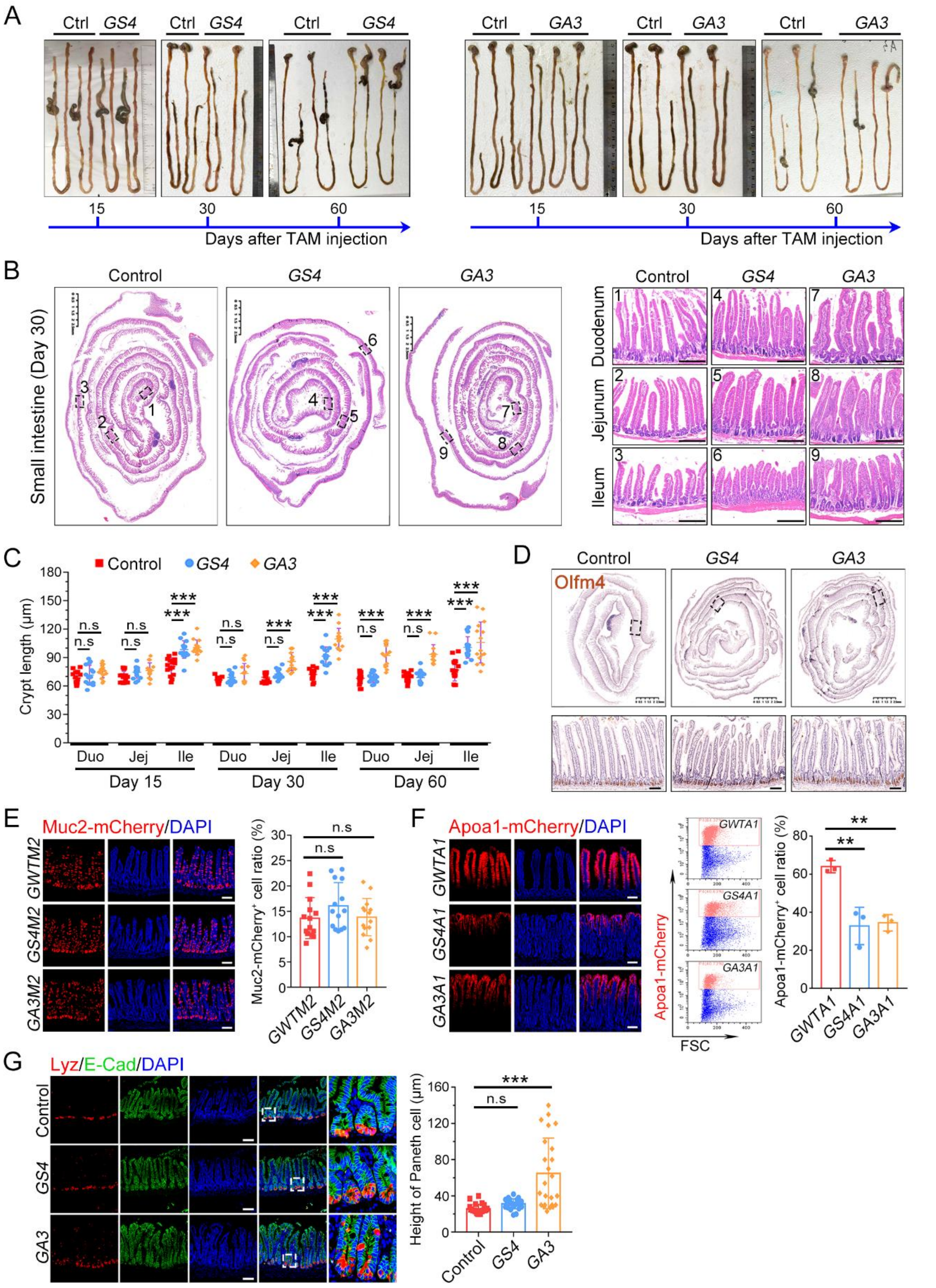

Fig. S2. Inactivation of *Smad4* or *Alk3* in Gli1^{+} stromal cells affects the differentiation of enterocytes and Paneth cells in the ileum.

(A) Representative images of small intestine at different time points post tamoxifen

administration. Ctrl, control.

(B) Representative images of H&E-stained small intestine sections and enlarged fields of three segments at 30 dpt. Scale bars: 2.5 mm (left), 200 µm (right).

(C) Crypt length of three small intestine segments at different times of conditional knockout. Duo: duodenum, Jej: jejunum, Ile: ileum.

(D) Representative images of Olfm4-stained small intestine sections and enlarged fields at 30 dpt. Scale bars: 2.5 mm (top), 100 µm (bottom).

(E) Representative images of Muc2-mCherry *in vivo* signals in ileum sections at 30 dpt, and the positive cell ratio was calculated against the total cell number. Scale bars: 100 µm.

(F) Representative images of Apoa1-mCherry signals in the ileum at 30 dpt, and the positive cell ratio was quantified by FACS analysis. Scale bars: 100 µm.

(G) Lyz and E-cadherin immunofluorescence staining of ileum sections at 30 dpt. The right panel shows the height of Paneth cell along the crypt. Scale bars: 100 µm.

n.s: no significance, $**P<0.01$, $***P<0.001$ by one-way ANOVA test in (C), (E), (F) and (G).

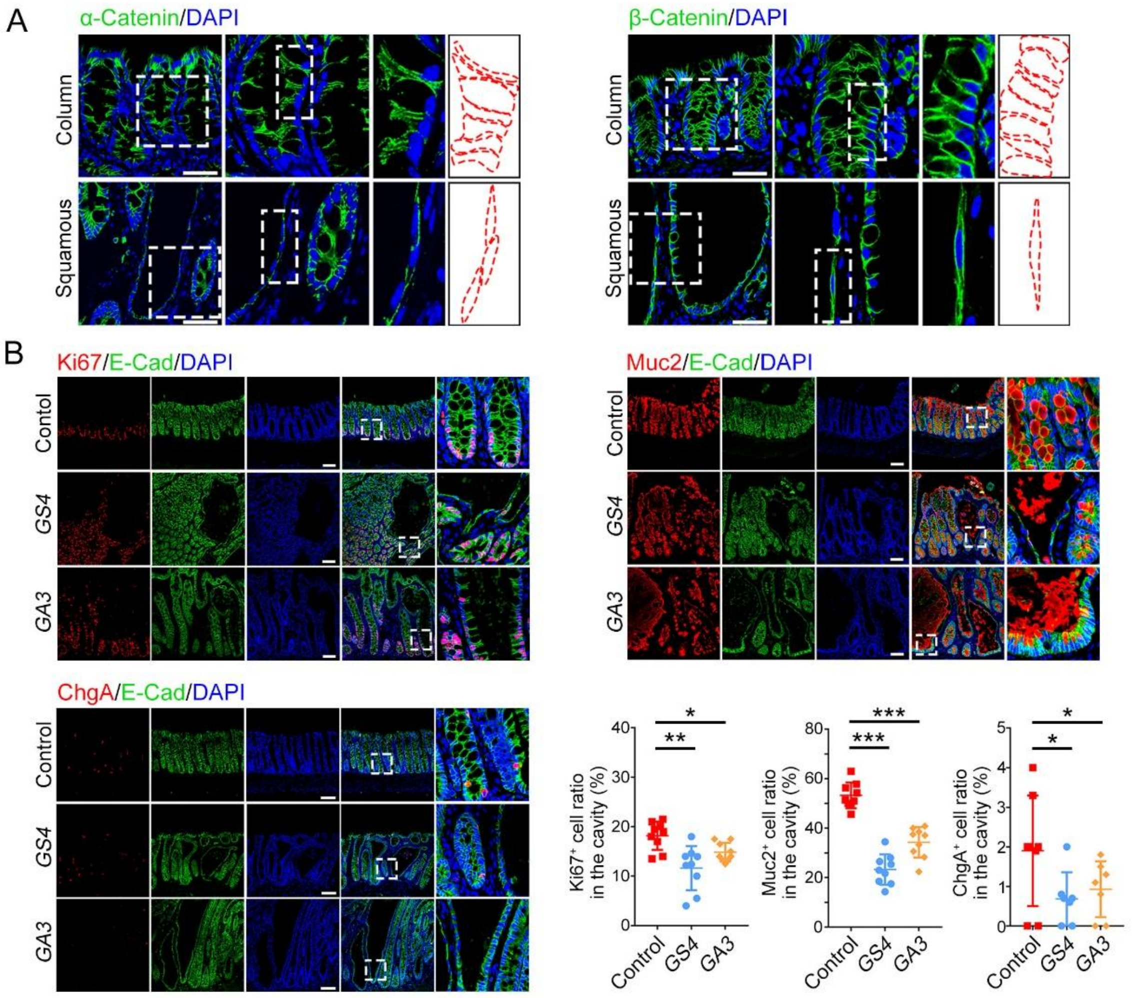

Fig. S3. Mucin accumulation leads to the morphological changes and cell ratio of epithelial cells in the colon.

(A) α-Catenin and β-catenin immunofluorescence staining in normal colon crypts and mucin-accumulated cavities. The right drawings depict the cell shape. Scale bars: 50 μm.

(B) Ki67, Muc2 and ChgA immunofluorescence staining of the epithelium around the cavities in distal colon sections at 30 dpt, and the positive cell ratio was calculated against the total cell number. Scale bars: 100 μm.

*P<0.05, **P<0.01, ***P<0.001 by one-way ANOVA test in (B).

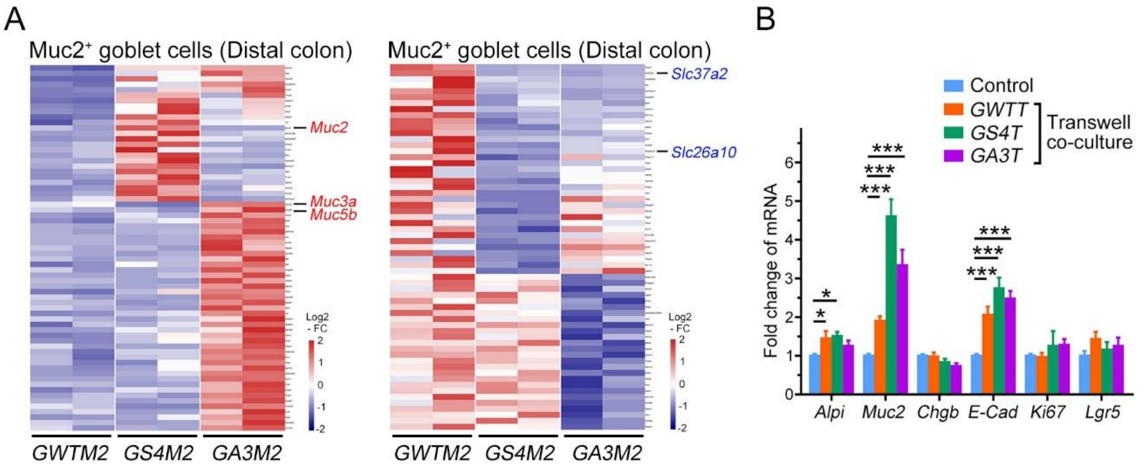

Fig. S4. Inactivation of *Smad4* or *Alk3* in Gli1$^+$ stromal cells alters the gene expression of goblet cells in the distal colon.

(A) Heatmap of the upregulated mucin-related genes (red) and the downregulated ion transport-related genes (blue) in Muc2-mCherry$^+$ cells from the indicated mice.

(B) qRT-PCR analysis of epithelial marker genes in distal colon organoids co-cultured with Gli1$^+$ cells from the indicated mice. 300 organoids were co-cultured with 10,000 Gli1-tdTomato$^+$ cells.

*P<0.05, ***P<0.001 by two-way ANOVA followed by Tukey's multiple comparisons test in (B).

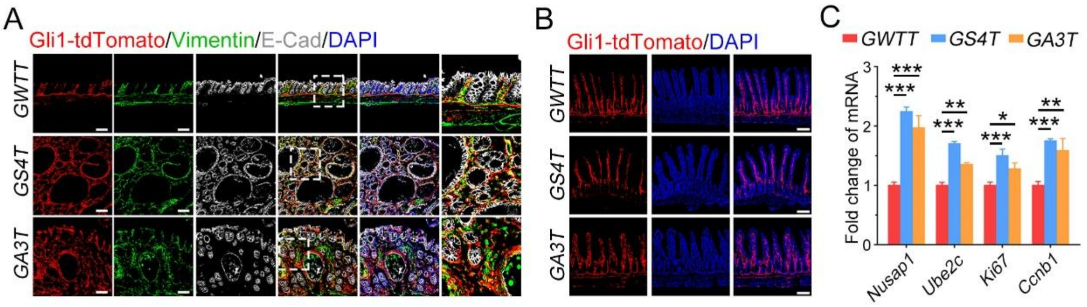

Fig. S5. BMP signaling inhibits proliferation of Gli1$^+$ stromal cells.

(A) Representative images of vimentin and E-cadherin immunofluorescence co-staining in Gli1-tdTomato distal colon sections at 30 dpt. Scale bars: 100 μm.

(B) Representative images of Gli1-tdToamto *in vivo* signals in small intestine sections at 30 dpt. Scale bars: 100 μm.

(C) qRT-PCR analysis of proliferation-related genes *in vitro* cultured Gli1-tdToamto$^+$ cells from the indicated mice.

*P<0.05, **P<0.01, ***P<0.001 by two-way ANOVA followed by Tukey's multiple comparisons test in (C).

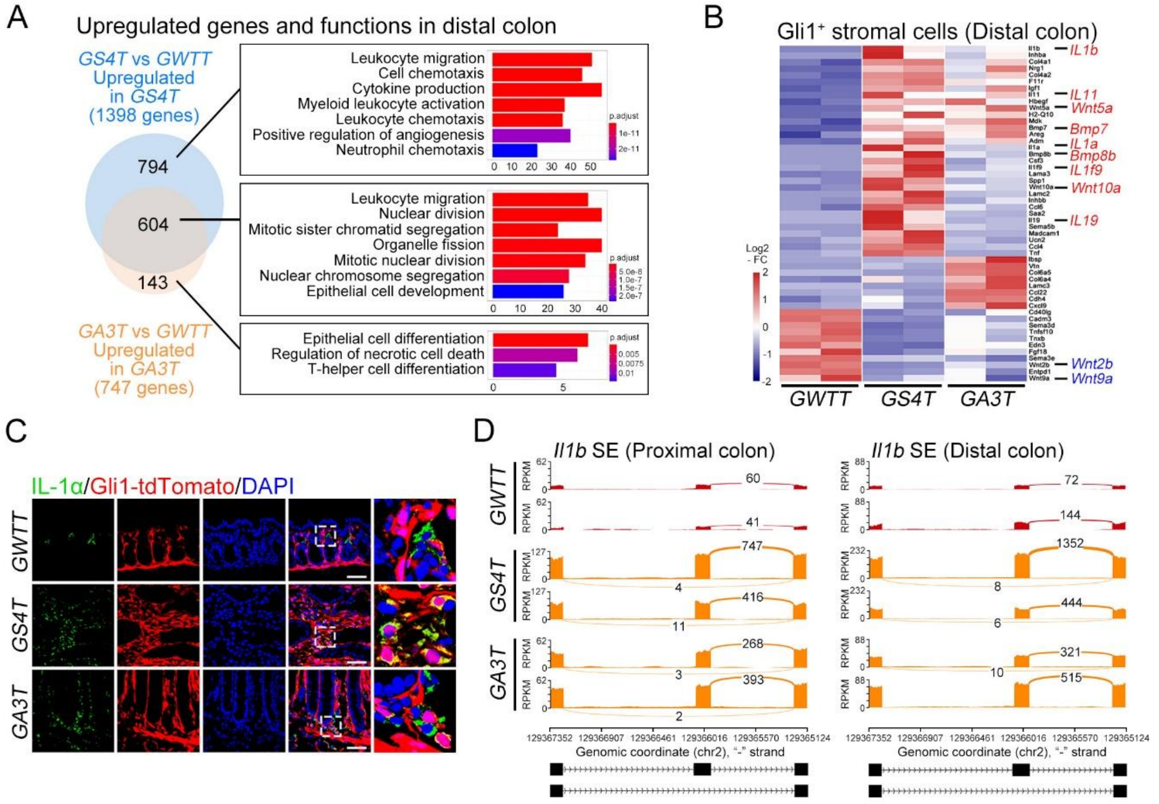

Fig. S6. BMP signaling enhances the interleukin production in Gli1^{+} stromal cells.

(A) GO analysis of the up-regulated genes in Gli1-tdToamto^{+} cells in distal colon from *GS4T* and *GA3T* mice compared with *GWTT* mice.

(B) Heatmap of the upregulated genes (Red) and the downregulated genes (Blue) in Gli1-tdToamto^{+} cells in distal colon from the indicated mice.

(C) Representative images of IL-1α immunofluorescence in Gli1-tdTomato distal colon sections at 30 dpt. Scale bars: 100 μm.

(D) Skipped exon (SE) analysis of *Il1b* in Gli1-tdToamto^{+} cells from the indicated mice.

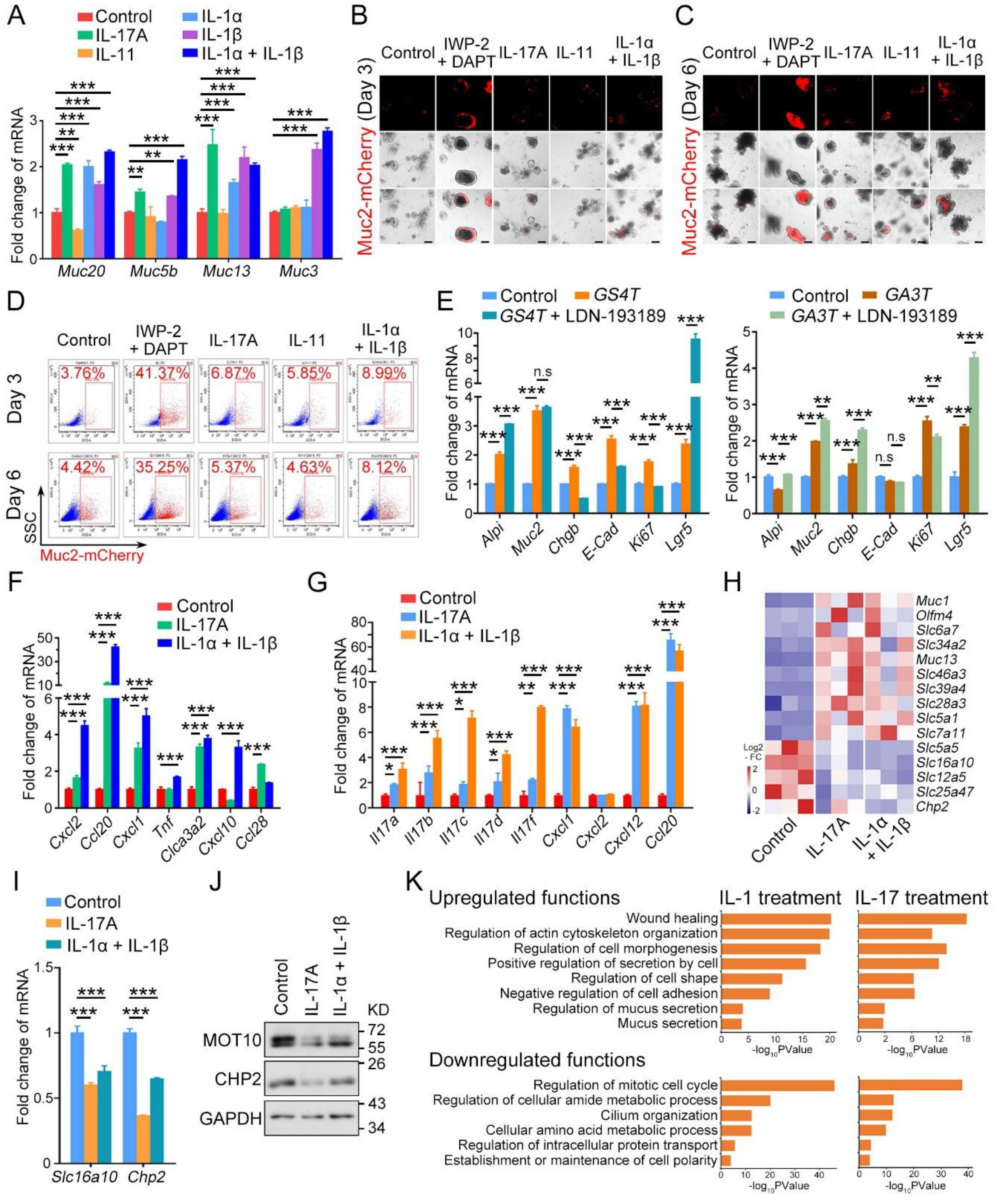

Fig. S7. IL-1 promotes the differentiation of goblet cells and regulates ion transport activities.

(A) qRT-PCR analysis of mucin-related genes in distal colon organoids at day 3 after treated with 10 nM interleukins.

(B) Representative images of Muc2-mCherry small intestinal organoids at day 3 after treated with 2 μM IWP-2, 10 μM DAPT or 10 nM interleukins. Scale bars: 100 μm.

(C) Representative images of Muc2-mCherry small intestinal organoids at day 6 after treated with 2 μM IWP-2, 10 μM DAPT or 10 nM interleukins. Scale bars: 100 μm.

(D) FACS analysis and quantitative results of Muc2-mCherry$^+$ cells from small intestinal organoids at day 3 or day 6 after treated with 2 μM IWP-2, 10 μM DAPT or 10 nM interleukins.

(E) qRT-PCR analysis of epithelial marker genes in distal colon organoids co-cultured with Gli1-tdToamto$^+$ cells from *GS4T* or *GA3T* at day 3 after treated with 1 μM LDN-193189.

(F) qRT-PCR analysis of upregulated genes from RNA-seq data in distal colon organoids after treated with 10 nM interleukins for 12 hours.

(G) qRT-PCR analysis of IL-17 target genes in distal colon organoids after treated with 10 nM interleukins for 72 hours.

(H) Heatmap of the function-related genes in distal colon organoids at day 3 after treated with 10 nM interleukins.

(I) qRT-PCR analysis of *Slc16a10* and *Chp2* in distal colon organoids at day 3 after treated with 10 nM interleukins.

(J) Immunoblot of protein MOT10 and CHP2 in distal colon organoids at day 3 after treated with 10 nM interleukins.

(K) GO analysis for the upregulated and downregulated functions at day 3 after the indicated treatments.

n.s: no significance, **P<0.01, ***P<0.001 by two-way ANOVA followed by Tukey's multiple comparisons test in (A), (E), (F), (G) and (I).

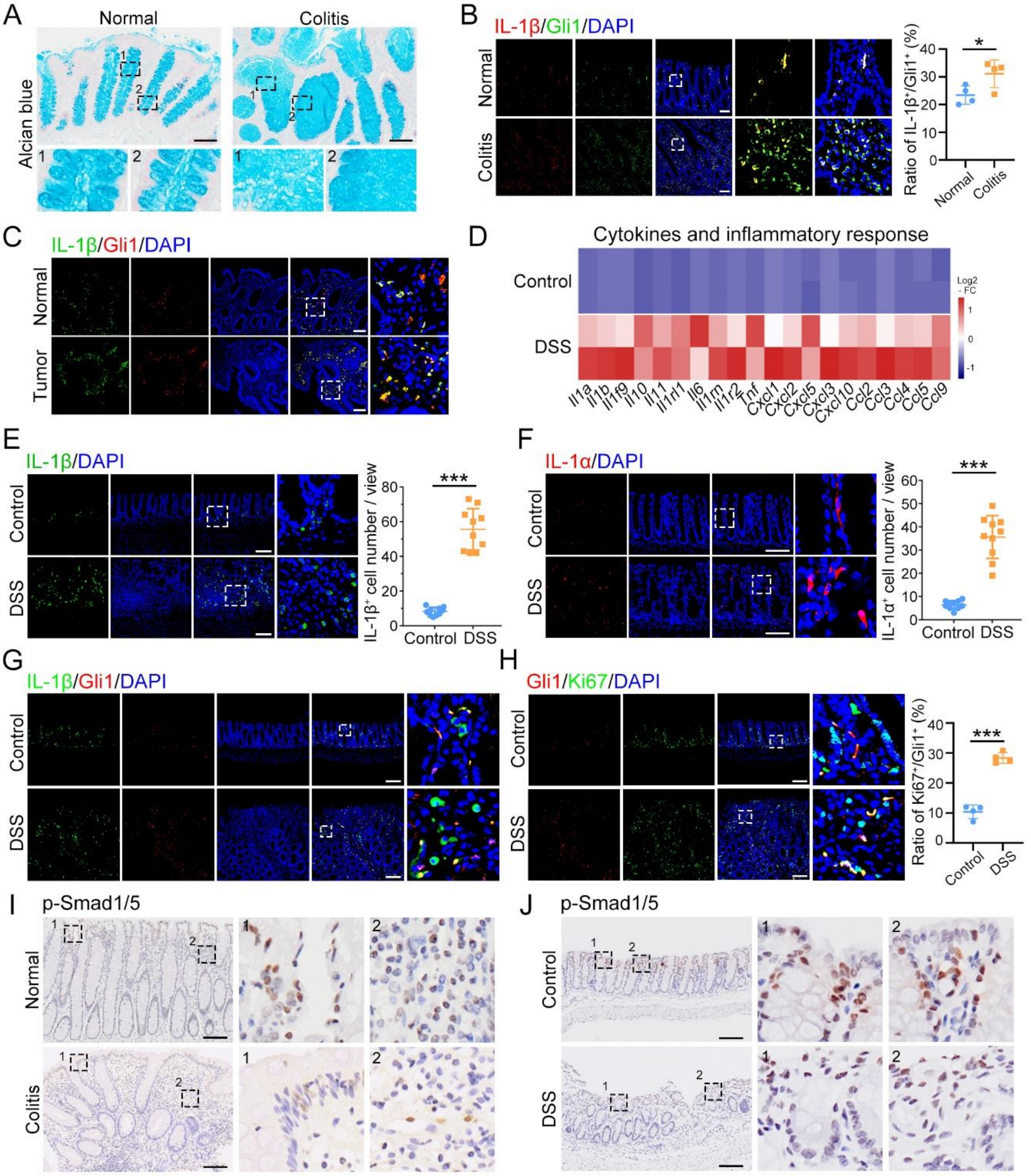

Fig. S8. IL-1 signaling is upregulated in DSS-induced colitis.

(A) Alcian blue staining of mucins in human large intestine and colitis sections. Scale bars: 100 μm.

(B) IL-1β and Gli1 immunostaining and quantification of human colon derived from healthy and ulcerative colitis specimens. Scale bars: 100 μm.

(C) Representative co-staining of IL-1β and Gli1 in human large intestine and colorectal cancer sections. Scale bars: 100 μm.

(D) Heatmap of the upregulated genes in colon from control and DSS-treated mice treated with 3% DSS in drinking water for 5 days.

(E) IL-1β immunofluorescence staining and quantification of IL-1β$^+$ cells (right) of colonic sections derived from control and DSS-treated mice. Scale bars: 100 μm.

(F) IL-1α immunofluorescence staining and quantification of IL-1α$^+$ cells (right) of colonic sections derived from control and DSS-treated mice. Scale bars: 100 μm.

(G) IL-1β and Gli1 immunostaining of large intestine derived from control and DSS-treated mice. Scale bars: 100 μm.

(**H**) Gli1 and Ki67 immunofluorescence staining and quantification of large intestine from control and DSS-treated mice. Scale bars: 100 μm.

(**I**) Representative images of p-Smad1/5 immunohistochemical staining in human large intestine and colitis sections. Scale bars: 100 μm.

(**J**) Representative images of p-Smad1/5 immunohistochemical staining of large intestine derived from control and DSS-treated mice. Scale bars: 100 μm.

***P<0.001 by one-way ANOVA test in (B), (E), (F), and (H).

Table S1 to S4

Table S1. Primer list for qRT-PCR

Table S2. RNA-seq results of Muc2-mCherry positive cells

Table S3. RNA-seq results of Gli1-tdTomato positive cells

Table S4. RNA-seq results of IL-1 or IL-17 treated organoids

Movie S1 to S3

Movie S1. The 3D structure of normal crypts revealed by Micro-CT

Movie S2. The 3D structure of cavities revealed by Micro-CT

Movie S3. Cavities in intestinal mucosa revealed by Micro-CT
